# Phenotypic plasticity of stomatal and photosynthetic features of four *Picea* species in two contrasting common gardens

**DOI:** 10.1093/aobpla/plz034

**Published:** 2019-06-08

**Authors:** Ming Hao Wang, Jing Ru Wang, Xiao Wei Zhang, Ai Ping Zhang, Shan Sun, Chang Ming Zhao

**Affiliations:** 1 State Key Laboratory of Grassland Agro-Ecosystems, School of Life Sciences, Lanzhou University, Lanzhou, Gansu, China; 2 Yuzhong Mountain Ecosystem Field Observation and Research Station, Lanzhou University, Lanzhou, Gansu, China; 3 Forestry College, Gansu Agricultural University, Lanzhou, Gansu, China

**Keywords:** Climate change, phenotypic plasticity, photosynthetic features, *Picea*, stomatal features

## Abstract

Global climate change is expected to affect mountain ecosystems significantly. Phenotypic plasticity, the ability of any genotype to produce a variety of phenotypes under different environmental conditions, is critical in determining the ability of species to acclimate to current climatic changes. Here, to simulate the impact of climate change, we compared the physiology of species of the genus *Picea* from different provenances and climatic conditions and quantified their phenotypic plasticity index (PPI) in two contrasting common gardens (dry vs. wet), and then considered phenotypic plastic effects on their future adaptation. The mean PPI of the photosynthetic features studied was higher than that of the stomatal features. Species grown in the arid and humid common gardens were differentiated: the stomatal length (SL) and width (SW) on the adaxial surface, the transpiration rate (Tr) and leaf mass per area (LMA) were more highly correlated with rainfall than other traits. There were no significant relationships between the observed plasticity and the species’ original habitat, except in *P*. *crassifolia* (from an arid habitat) and *P. asperata* (from a humid habitat). *Picea crassifolia* exhibited enhanced instantaneous efficiency of water use (PPI = 0.52) and the ratio of photosynthesis to respiration (PPI = 0.10) remained constant; this species was, therefore, considered to the one best able to acclimate when faced with the effects of climate change. The other three species exhibited reduced physiological activity when exposed to water limitation. These findings indicate how climate change affects the potential roles of plasticity in determining plant physiology, and provide a basis for future reforestation efforts in China.

## Teaser

The four species in the genus of *Picea* analysed in this study exhibited comparatively high photosynthetic plasticity and low stomatal plasticity in the two common gardens. The results also indicated that site-specific conditions can mask habitat variations, and thus should always be considered in studies of habitat trends in the future. Finally, we can make a preliminary suggestion that *P. crassifolia* should survive best. These findings indicate how climate change affects the potential roles of plasticity in determining plant physiology, and provide a basis for future reforestation efforts in China.

## Introduction

The global climate will change significantly over the coming century, with overall increases in temperature and a greater frequency of drought events in many regions (Gitay *et al.* 2002). This will affect the exchange of water, carbon, nutrients, and energy between plants and the environment, particularly in mountain ecosystems (IPCC 2007; Grossiord *et al.* 2017a). Previous research indicates that plant species can adjust to climate change through phenotypic plasticity—the range of phenotypes that a genotype can express as a function of the range of environmental conditions. However, the range of variation within genotypes varies depending on the scale of the particular study (species, population, etc.) (Miner *et al.* 2005; Nicotra *et al.* 2010; Pigliucci 2010). In the context considered here, we can hypothesize that species may have the greatest capacity for acclimation as a result of increased phenotypic plasticity. Generally, species may be constrained by the selective pressures acting in their particular native climate. So the species exposed to more climatic variation in their distribution range are reported to have more phenotypic plasticity than species exposed to restricted climatic variation in their ranges (Valladares *et al.* 2014). Therefore, it is valuable to quantify a species’ ability to rapidly adjust functional traits, both morphological and physiological (Grulke 2010; Nicotra *et al.* 2010; Frank *et al.* 2017).

The growth and survival of plants during periods of drought depends on those traits affecting the absorption, transport and conservation of water; these include stomatal regulation of water loss, osmotic adjustment of the leaf turgor loss point, and the structure of the xylem and the roots (Brodribb and Holbrook 2003; McDowell *et al.* 2008; Bartlett *et al.* 2016; Pfautsch *et al.* 2016). Moreover, stomata regulate gas exchange, a key process enabling plants to cope with rapid climate change (Nicotra *et al.* 2010; Grossiord *et al.* 2017a). Stomatal structure and behaviour have particularly important effects on water loss, hydraulic characteristics and carbon acquisition, and have therefore been studied in several plant species (Brodribb and Holbrook 2003; Choat *et al.* 2011; Bourne *et al.* 2017). For instance, Brodribb *et al.* (2003) demonstrated that the point of stomatal closure occurs prior to cavitation and relates to stem vulnerability and the water potential at the turgor loss point in eight tropical dry forest trees. In addition, Wang *et al.* (2007) concluded that the density, size and distribution of the stomata all had important effects on water exchange between plants and their environment. There is also evidence that stomatal density increases and/or stomatal size decreases in more arid climates and at higher temperatures (Franks and Farquhar 2001; Zhao *et al.* 2015; Carlson *et al.* 2016). Leaf mass per unit area and water use efficiency are key adaptive plastic traits for assessing plants’ responses to climate change (Hetherington and Woodward 2003; Wright *et al.* 2004; Seibt *et al.* 2008). However, it is not clear how the plasticity of stomatal density and size relate to the plasticity of gas exchange capacity in arid climates (Locosselli and Ceccantini 2012), especially when focusing on plants belonging to a single genus.

*Picea* species, which are important components of the alpine and subalpine coniferous forests of the Northern Hemisphere, may be exposed to frequent drought events as a result of climate change. *Picea meyeri*, *P. crassifolia*, *P. asperata* and *P. wilsonii* are species endemic to China (Fu *et al.* 1999), and climate change is expected to make the regions in which they are found drier than they are at present (Fu and Ma 2008; Huang *et al.* 2016). Coincidentally, the mean annual precipitation in the native ranges of *P*. *meyeri* and *P*. *crassifolia* is lower (491 and 417 mm, respectively) than that in the native ranges of *P*. *asperata* and *P*. *wilsonii* (714 and 690 mm, respectively). Therefore, the existence of four *Picea* species from contrasting climates provides us with an opportunity to evaluate the phenotypic plasticity of stomatal and photosynthetic features in spruce saplings under simulated climate change scenarios. Generally, species that originate from arid regions may benefit from maintaining a low stomatal conductance and increasing water use efficiency by minimizing transpiration (Bourne *et al.* 2017). Conversely, plants from humid areas may benefit from high rates of assimilation and productivity (Fichot *et al.* 2009). Therefore, we gathered seeds from a site at the centre of the distribution range of each of the four species and them reared in a greenhouse, then planted the saplings into two different common gardens (wet and dry), where confounding environmental and phylogenetic factors were minimized, to simulate the impact of climate change. Stomatal anatomic traits were measured separately on the adaxial and abaxial surface due to their differing morphology, according to classical taxonomy (Fu *et al.* 1999). To assess the functional status of the stomata, photosynthetic features were also measured, including net photosynthetic rate (Pn), the instantaneous efficiency of water use (iWUE) and photosynthesis/respiration ratio (P/R), which can be used to evaluate the balance of water loss and carbon acquisition. Finally, we aimed to investigate the degree and relationships between stomatal and photosynthetic plasticity of the four *Picea* species and the relationship between their original habitats. More specifically, we expect the species from dry habitats to achieve higher plasticity with respect to regulating water loss, whilst species from humid habitats could be expected to exhibit greater variability in physiological traits related to productivity. Furthermore, a better understanding of the phenotypic plasticity of *Picea* species will assist in compiling guidelines for future reforestation efforts in China and elsewhere.

## Materials and Methods

### Study site and experimental design

One of the common gardens is in the southeast of Gansu province, at Zhangjia forest station in Liangdang County (106.5575°E, 34.1186°N; WCG denoting the wet common garden). This region has a medium latitude monsoon climate, with a mean annual precipitation and temperature of 754 mm and 7.8 °C, respectively. The other common garden is in the middle of Gansu province, at the Plant Germplasm Repository of Lanzhou University in Yuzhong County (104.1562°E, 55.9391°N; DCG denoting the dry common garden). This region has a temperate semi-arid climate, with a mean annual precipitation and temperature of 385 mm and 7.1 °C, respectively. The two sites thus have similar annual temperatures but different levels of precipitation, making them suitable for simulating the effect of a transition from a wet climate to a dry one.

Seeds of each species (*Picea meyeri*, *P*. *crassifolia*, *P*. *asperata* and *P*. *wilsonii*) were collected from their respective central distribution ranges during 2004 and 2005. As noted above, the habitats of *P*. *crassifolia* and *P*. *meyeri* have lower mean annual precipitation levels than those of *P*. *asperata* and *P*. *wilsonii*. All seeds were germinated at a tree nursery and grown on until they were 5 years old, after which saplings were successively transplanted to the two study sites in the spring of 2010. Planting consisted of four blocks (replicates); seedlings from each species were planted 1 m apart in 3 × 3 square matrices (9 individuals per block), and randomly assigned within each block (Oleksyn *et al.* 1998).

### Anatomy of stomatal features

**Figure 1.**
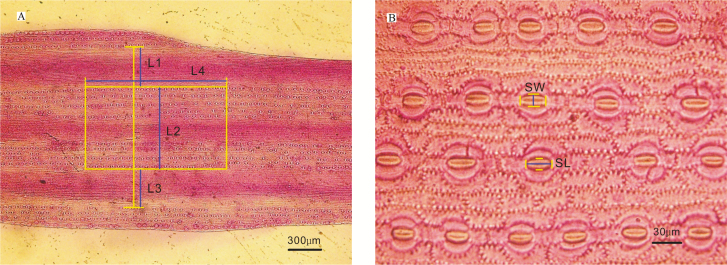
Micrographs of the adaxial epidermis of *Picea crassifolia* at (A) ×40 and (B) ×400 magnification. (A) L1 and L3 are the distances between adaxial and abaxial stomatal rows. L2 is the distance between stomata on the adaxial surface. L4 is the width to stomatal number on this surface. Stomatal density (SD) was calculated as: stomatal number/{[(L1 + L2)/2 + L3] × L4}. (B) SL (stomatal length) and SW (stomatal width) are measured directly. Bars = 300 µm (A), 30 µm (B).

Ten individuals per species were randomly chosen from four blocks for anatomical measurements. Current-year needles were collected in mid-September 2013 to ensure that the collected leaves were fully expanded and mature. On a sunny morning, current-year twigs all with the same orientation were cut down and immediately placed in 10-mL centrifuge tubes containing modified FAA solution (50 % alcohol, formalin, glacial acetic acid and glycerinum in a volume ratio of 90:5:5:5) to prevent leaf deformation and stomatal closure. All samples were then taken back to the laboratory for the measurement of anatomical characteristics. Twenty needles from each twig were randomly selected and dissociated in sodium hypochlorite (10 % available chlorine). Because needles of spruce are quadrangular in cross section with four ridges, we sliced the mid-ridge of one side to distinguish the adaxial and abaxial surface and also to allow easy dissociation of the mesophyll tissue in sodium hypochlorite. After immersion for 8 h, a banister brush was used carefully to clean the residual mesophyll cells without destroying the epidermis. The transparent epidermis pieces were then dyed with safranine (1 %) to make temporary sections, which were imaged using an Olympus photomicroscope (BX51, Olympus (China) Co., Ltd) at ×40 and ×400 magnification (Fig. 1, an example of *P*. *crassifolia*).

Stomatal density (SD, mm^−2^) values were calculated as the total number of stomata per 1 mm^2^ area on the adaxial or abaxial surface. The number of stomatal rows (N) was manually counted using the ×40 images (incomplete rows were assigned a value of 0.5), while stomatal counts on the epidermis were conducted near the middle of the needle on the same surface to avoid potential variation at the base or tip (Fig. 1A). The ×400 images were used to determine the stomatal pore length (SL, μm) and width (SW, μm) by measuring the longest axis and the widest point perpendicular to this (Fig. 1B). The mean stomatal pore area (S, μm^2^) was defined as an ellipse with major axis equal to SL and minor axis equal to SW, as described by Dow *et al.* (2013). Stomatal pore index (SI) was the pore area per leaf area and was calculated as SD multiplied by S. All image analysis was performed using the Image-Pro Plus 5.0.1 (Media Cybernetics, Inc., USA) software package.

### Measurement of photosynthetic features

We measured photosynthetic features on single current-year sun-exposed twigs of the selected individuals in mid-September. The features examined were the leaf mass per unit area (LMA, kg m^−2^), net photosynthesis rate (Pn, µmol cm^−2^ s^−1^), stomatal conductance (Gs, mol cm^−2^ s^−1^), transpiration rate (Tr, mmol cm^−2^ s^−1^), respiration rate during daytime darkness (Rday, mol cm^−2^ s^−1^), instantaneous efficiency of water use (iWUE, µmol mmol^−1^) and P/R (photosynthesis/respiration ratio). At least three randomly chosen twigs per selected individual in the four blocks were measured with a Li-6400XT infrared gas exchange system (LI-COR Biosciences, Lincoln, NE, USA). Measurements were taken in the morning (between 08:30 and 11:30 a.m.) when the highest stomatal conductance could be expected. Measurements were made using a 6400-22L conifer chamber (RGB sources) with a reference CO_2_ concentration of 380 µmol mol^−1^, a reference chamber temperature of 25 °C, a flow rate of 300 µmol s^−1^, and two light levels of 1000 (saturated light) and 0 μmol m^−2^ s^−1^ to obtain the maximum gas exchange and daytime respiration, respectively (see Drake *et al.* 2013). After that, all needles were scanned to measure their projected areas and then dried to constant mass (>48 h) in an oven at 72 °C to allow us to calculate LMA.

### Phenotypic plasticity index

For all studied traits, a phenotypic plasticity index (PPI) was calculated as described by Larcher *et al.* (2015) and Santiso and Retuerto (2015), using the following expression: PPI = (species mean 1 – species mean 2)/(the larger species mean). The index varies from 0 (no plasticity) to 1 (the maximum plasticity), and allows comparisons between traits with different units (Valladares *et al.* 2006; Larcher *et al.* 2015; Santiso and Retuerto 2015).

### Data analysis

A one-way ANOVA was used to test whether trait means differed significantly among species and between the two gardens; analysis was conducted using SPSS 16.0 (SPSS Inc., Chicago, IL, USA). Differences were considered statistically significant if *P* < 0.05. The PPI values were calculated in Excel after the analysis above. To understand how the stomatal and photosynthetic traits differed between species and study sites, and to describe the relationships between the studied traits, we performed a principal component analysis (PCA) using the Past 2.17c software (Hammer *et al.* 2001) after removing non-independent and highly correlated traits. Intraspecific comparisons between the two common gardens were performed via independent samples *t*-tests with SPSS 16.0 and variation trends for all traits were plotted using SigmaPlot 10.0 (Systat Software, Inc., Chicago, IL, USA).

## Results

### Differences in stomatal and photosynthetic features for species and sites

For stomatal features, significant differences were observed between adaxial and abaxial surfaces among the four *Picea* species from the two sites. That is, on the adaxial surface, *P. asperata* had the highest stomatal pore length (SL), stomatal pore width (SW), stomatal area (S) and stomatal pore index (SI); *P. crassifolia* exhibited the highest adaxial stomatal density (SD) and the lowest SL and S; and *P. wilsonii* had the lowest number of stomatal rows (N) among the four species when grown in WCG (Table 1). On the abaxial surface, *P. meyeri* exhibited the highest values of all stomatal traits except for N and SL, *P. wilsonii* had the lowest SD and N, and *P. crassifolia* had the lowest SL and S among the four species when grown in WCG (Table 1). However, in DCG, these interspecific differences on the adaxial surface were different: *P. meyeri* exhibited the highest SD and SI, and *P. crassifolia* tended to have the highest S on the adaxial surface among the four species (Table 2). However, the interspecific differences on the abaxial surface in DCG were similar to those found in WCG: *P. meyeri* had the highest S and SI and *P. wilsonii* tended to have the lowest SD and N among the four species (Tables 1 and 2).

**Table 1.** Stomatal and photosynthetic features of four *Picea* species grown in WCG. Row legends: SD (stomatal density); N (number of stomatal rows); SL (stomatal pore length); SW (stomatal pore width) and S (stomatal pore area); SI (stomatal pore index); Pn (net photosynthetic rate); Gs (stomatal conductance); Tr (transpiration rate); Rday (respiration rate during daytime darkness); iWUE (instantaneous efficiency of water use); P/R (photosynthesis/respiration ratio); LMA (leaf mass per area). Data are presented as mean ± SE. Significance of interspecies differences is based on one-way ANOVA and marked with A, B and C.

Features		*P. meyeri*	*P. crassifolia*	*P. asperata*	*P. wilsonii*
SD (mm^−2^)	Adaxial	42 ± 3B	53 ± 3A	38 ± 0B	40 ± 2B
	Abaxial	32 ± 1A	30 ± 0B	30 ± 1B	23 ± 1C
N	Adaxial	3.6 ± 0.2A	3.8 ± 0.1A	3.4 ± 0.2A	2.9 ± 0.1B
	Abaxial	2.8 ± 0.1A	2.6 ± 0.2A	2.6 ± 0.1A	1.5 ± 0.1B
SL (μm)	Adaxial	34 ± 1B	27 ± 1C	37 ± 1A	34 ± 1B
	Abaxial	35 ± 0A	28 ± 1B	34 ± 1A	34 ± 0A
SW (μm)	Adaxial	15 ± 0.4BC	14 ± 0.5C	18 ± 0.7A	16 ± 0.1B
	Abaxial	18 ± 0.7A	14 ± 0.2C	15 ± 0.3B	14 ± 0.1BC
S (μm^2^)	Adaxial	410 ± 13B	299 ± 16C	520 ± 26A	422 ± 9B
	Abaxial	484 ± 16A	301 ± 4C	409 ± 12B	387 ± 6B
SI	Adaxial	0.017 ± 0.001AB	0.016 ± 0.002B	0.02 ± 0.001A	0.017 ± 0.000AB
	Abaxial	0.016 ± 0.001A	0.009 ± 0.000C	0.012 ± 0.001B	0.009 ± 0.000C
Pn (µmol cm^−2^ s^−1^)		9.4 ± 0.5A	7.8 ± 0.2B	6.7 ± 0.7B	6.8 ± 0.3B
Gs (mol cm^−2^ s^−1^)		0.11 ± 0.01A	0.09 ± 0.00AB	0.08 ± 0.01B	0.07 ± 0.00B
Tr (mmol cm^−2^ s^−1^)		2.1 ± 0.25A	2.0 ± 0.19A	1.4 ± 0.15B	2.1 ± 0.13A
Rday (µmol cm^−2^ s^−1^)		−1.1 ± 0.1A	−1.8 ± 0.3B	−1.1 ± 0.1A	−0.8 ± 0.1A
iWUE (µmol mmol^−1^)		4.7 ± 0.8A	3.9 ± 0.5AB	4.7 ± 0.2AB	3.2 ± 0.1B
P/R		8.5 ± 0.7A	6.4 ± 1.4AB	4.6 ± 0.9B	9.1 ± 0.5A
LMA (kg m^−2^)		0.19 ± 0.00AB	0.20 ± 0.02A	0.22 ± 0.01A	0.16 ± 0.01B

**Table 2.** Stomatal and photosynthetic features of four *Picea* species grown in DCG. Data are presented as mean ± SE. Significance of interspecies differences is based on one-way ANOVA and marked with A, B and C.

Features		*P. meyeri*	*P. crassifolia*	*P. asperata*	*P. wilsonii*
SD (mm^−2^)	Adaxial	47 ± 2A	39 ± 2B	37 ± 1B	35 ± 1B
	Abaxial	32 ± 1A	32 ± 2A	30 ± 1A	22 ± 1B
N	Adaxial	4.3 ± 0.1A	4.1 ± 0.3A	3.4 ± 0.1B	3.0 ± 0.1B
	Abaxial	3.3 ± 0.1B	3.8 ± 0.1A	3.1 ± 0.0B	2.0 ± 0.1C
SL (µm)	Adaxial	36 ± 1B	38 ± 1A	35 ± 1B	40 ± 1A
	Abaxial	37 ± 0A	34 ± 1B	30 ± 1C	36 ± 1A
SW (µm)	Adaxial	19 ± 0.4A	19 ± 0.7A	19 ± 0.4A	17 ± 0.1B
	Abaxial	20 ± 0.3A	14 ± 0.4B	14 ± 0.9B	15 ± 0.5B
S (µm^2^)	Adaxial	548 ± 10B	579 ± 8A	507 ± 6C	521 ± 9BC
	Abaxial	571 ± 9A	386 ± 21BC	333 ± 13C	423 ± 23B
SI	Adaxial	0.026 ± 0.001A	0.023 ± 0.001B	0.019 ± 0.000C	0.018 ± 0.001C
	Abaxial	0.018 ± 0.001A	0.012 ± 0.001B	0.01 ± 0.001C	0.009 ± 0.000C
Pn (µmol cm^−2^ s^−1^)		7.1 ± 0.1AB	7.5 ± 0.6A	6.9 ± 0.3AB	6.2 ± 0.2B
Gs (mol cm^−2^ s^−1^)		0.07 ± 0.004B	0.10 ± 0.003A	0.07 ± 0.003B	0.07 ± 0.002B
Tr (mmol cm^−2^ s^−1^)		1.3 ± 0.0B	1.6 ± 0.1A	0.9 ± 0.1C	1.7 ± 0.1A
Rday (µmol cm^−2^ s^−1^)		−1.9 ± 0.3A	−2.0 ± 0.2A	−1.4 ± 0.1A	−2.0 ± 0.4A
iWUE (µmol mmol^−1^)		5.6 ± 0.2B	4.9 ± 0.3B	8.2 ± 0.6A	3.6 ± 0.3C
P/R		4.1 ± 0.8AB	3.7 ± 0.3AB	5.1 ± 0.4A	3.2 ± 0.5B
LMA (kg m^−2^)		0.27 ± 0.02B	0.34 ± 0.01A	0.36 ± 0.01A	0.28 ± 0.01B

Significant differences were also observed in photosynthetic features among species. In WCG, *P. meyeri* showed the highest net photosynthetic rate (Pn) and stomatal conductance (Gs), while *P. asperata* tended to have the lowest transpiration rate; *P. crassifolia* had the lowest respiration rate during daytime darkness (Rday) and the lowest ratio of photosynthesis to respiration (P/R) (Table 1). In DCG, *P. asperata* had the highest Pn and Gs, *P. crassifolia* had the highest instantaneous efficiency of water use (iWUE) and lowest transpiration rate (Tr), while *P. wislonii* exhibited the lowest iWUE among the four species (Table 2).

### Plasticity of stomatal and photosynthetic features

**Figure 2.**
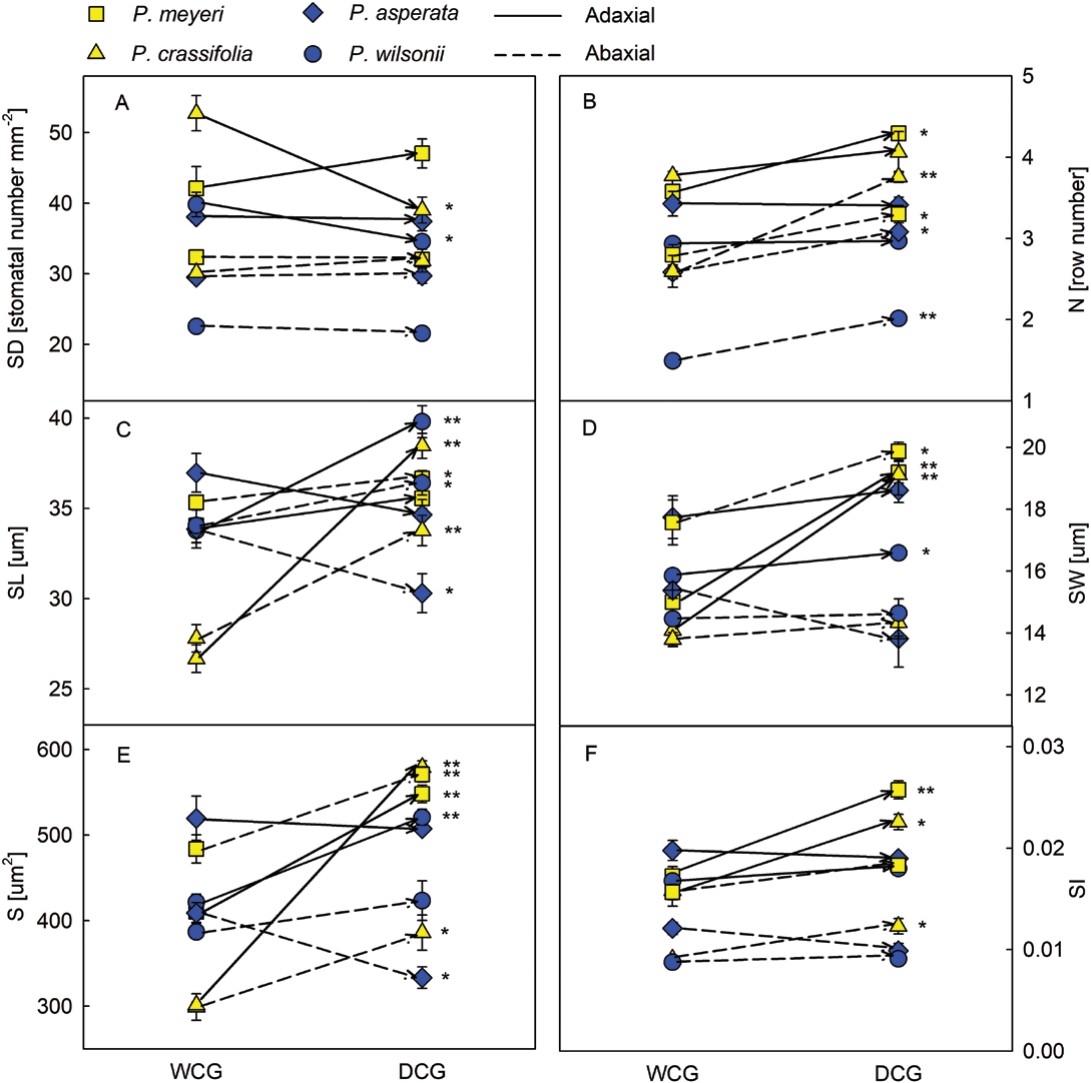
Differences in stomatal traits between WCG and DCG for each species. Yellow symbols indicate the species from the dry habitat and blue symbols indicate the species from the wet habitat. The lines show mean values and standard error of means across three replicates for each measurement, plotted against drought treatments. (A) SD is stomatal density; (B) N is number of stomatal rows; (C) SL is stomatal pore length; (D) SW is stomatal pore width; (E) S is stomatal pore area; (F) SI is stomatal pore index. *P < 0.05 and **P < 0.01.

**Figure 3.**
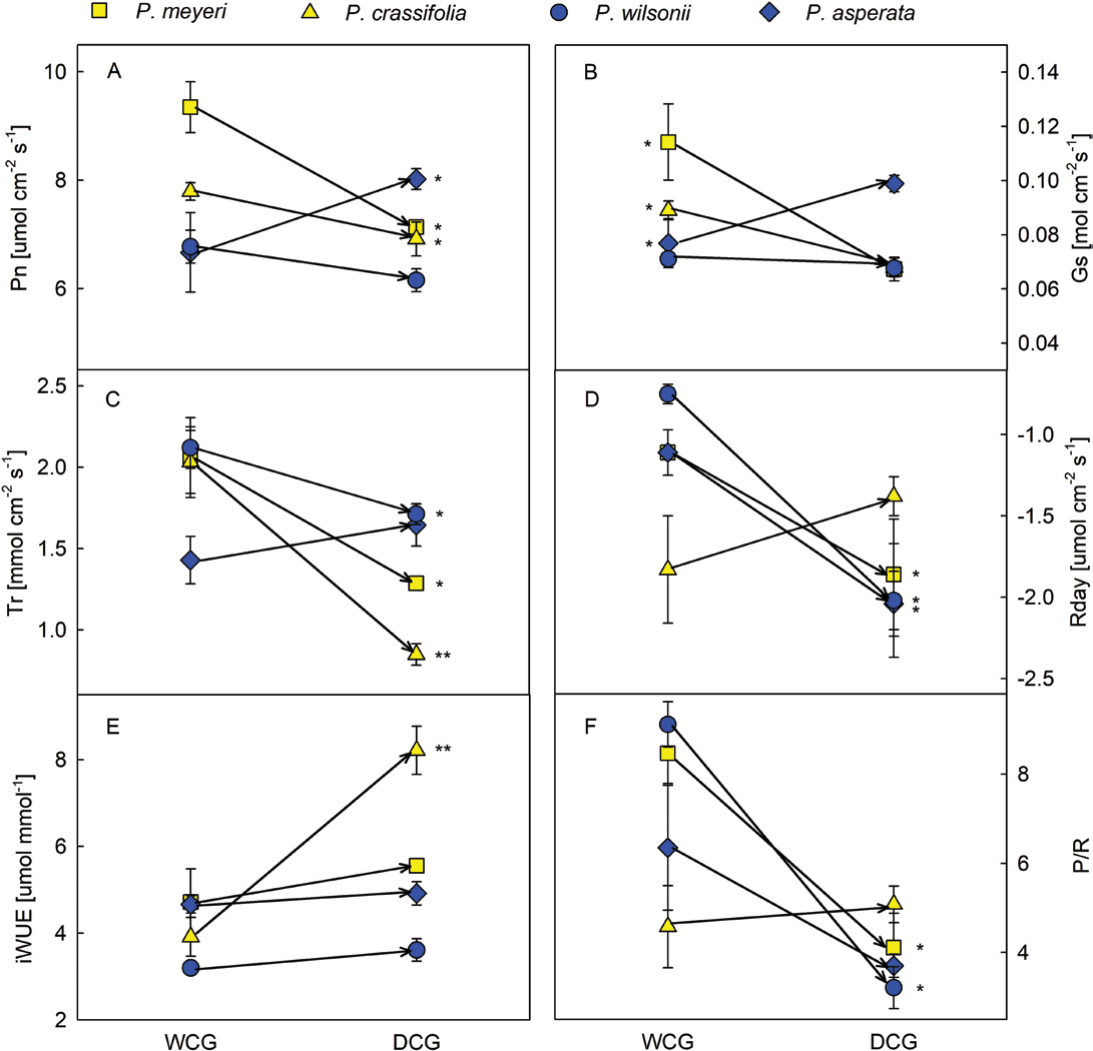
Differences in photosynthetic traits between WCG and DCG for each species. Yellow symbols indicate the species from the dry habitat and blue symbols indicate the species from the wet habitat. The lines show mean values and standard error of means across three replicates for each measurement, plotted against drought treatments. (A) Pn is net photosynthetic rate; (B) Gs is stomatal conductance; (C) Tr is transpiration rate; (D) Rday is respiration rate during daytime darkness; (E) iWUE is instantaneous efficiency of water use; (F) P/R is photosynthesis/respiration ratio. *P < 0.05 and **P < 0.01.

**Figure 4.**
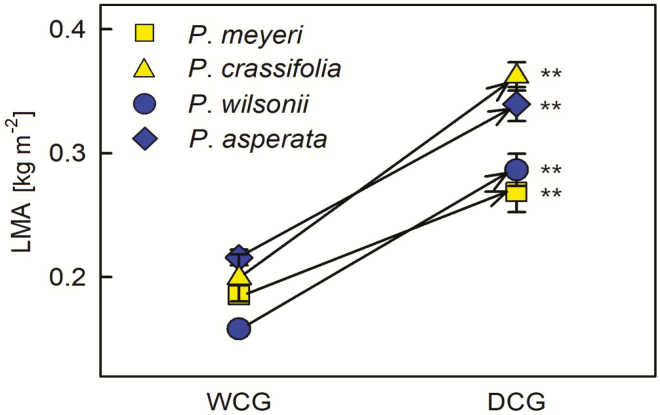
Differences in leaf mass per area (LMA) between WCG and DCG for each species. Yellow symbols indicate the species from the dry habitat and blue symbols indicate the species from the wet habitat. The lines show mean values and standard error of means across three replicates for each measurement, plotted against drought treatments. **P < 0.01.

These four species exhibited different plastic responses between the two sites with their different amounts of rainfall. That is, *P. meyeri* tended to have higher N, SW and S on both the adaxial and abaxial surfaces in DCG than in WCG, but its photosynthetic features (Pn, Gs, Tr and P/R) in DCG had lower values than in WCG (Figs 2 and 3; *P* < 0.05). Similarly, *P. crassifolia* exhibited higher SL, S and SI on both the adaxial and abaxial surfaces, as well as higher abaxial N, higher adaxial SW and higher iWUE in DCG than in WCG; while its adaxial SD, Gs and Tr in DCG were lower than in WCG (Figs 2 and 3; *P* < 0.05). *Picea wilsonii* also had higher SL on both the adaxial and abaxial surfaces, higher abaxial N and adaxial SW and S in DCG than in WCG, but its Tr, Rday and P/R values were lower in DCG than in WCG (Figs 2 and 3; *P* < 0.05). *Picea asperata* had higher abaxial N in DCG than in WCG, but its abaxial S and Rday in DCG were lower than in WCG (Figs 2 and 3; *P* < 0.05). Additionally, the leaf mass per area (LMA) of all species was higher in DCG than in WCG (Fig. 4). The correlation analysis indicated that SL and SW on the adaxial surface, Tr and LMA were correlated with many traits **[see****Supporting Information—Table S1****]**, which would imply that changes in these characteristics were important to species acclimation.

Overall, the morphological features of stomata exhibited limited plasticity: the mean PPI values of independent traits for all species were below 0.18 and the highest PPI value was observed in *P*. *crassifolia* (0.31); the non-independent traits, stomatal pore area (S) and stomatal pore index (SI) showed more plasticity in *P*. *meyeri* and *P*. *crassifolia* than in the other species (Table 3). The mean PPI values of photosynthetic features were higher than those of stomatal features (Table 3). In addition, the species that originated in arid regions (*P*. *meyeri* and *P*. *crassifolia*) exhibited higher gas exchange plasticity in terms of Gs and Tr than those which originated in humid habitats (*P*. *asperata* and *P*. *wilsonii*). In contrast, the plasticity of Rday in species from humid habitats was higher than in those from arid areas.

**Table 3.** Phenotypic plasticity index values and mean values of the four species. Mean PPI values with independent traits are calculated with traits of each category and highlighted in bold.

Traits		*P*. *meyeri*	*P*. *crassifolia*	*P*. *asperata*	*P*. *wilsonii*
SD (mm^−2^)	Adaxial	0.10	0.26	0.02	0.13
	Abaxial	0.01	0.05	0.01	0.04
N	Adaxial	0.17	0.07	0.00	0.01
	Abaxial	0.15	0.31	0.16	0.26
SL (µm)	Adaxial	0.04	0.31	0.06	0.15
	Abaxial	0.04	0.18	0.10	0.06
SW (µm)	Adaxial	0.22	0.26	0.05	0.04
	Abaxial	0.12	0.04	0.10	0.01
S (µm^2^)	Adaxial	0.25	0.48	0.02	0.19
	Abaxial	0.15	0.22	0.18	0.09
SI	Adaxial	0.33	0.30	0.04	0.07
	Abaxial	0.14	0.26	0.18	0.04
Mean PPI of adaxial traits		**0.13**	**0.23**	**0.03**	**0.23**
Mean PPI of abaxial traits		**0.08**	**0.14**	**0.09**	**0.10**
Mean PPI with independent traits		**0.11**	**0.18**	**0.06**	**0.09**
Pn (µmol cm^−2^ s^−1^)		0.24	0.11	0.11	0.09
Gs (mol cm^−2^ s^−1^)		0.41	0.24	0.22	0.05
Tr (mmol cm^−2^ s^−1^)		0.38	0.58	0.13	0.19
Rday (µmol cm^−2^ s^−1^)		0.40	0.25	0.46	0.67
iWUE (µmol mmol^−1^)		0.15	0.52	0.05	0.11
P/R		0.52	0.10	0.46	0.70
Mean PPI with independent traits		**0.36**	**0.29**	**0.23**	**0.25**
LMA (kg m^−2^)		0.31	0.45	0.36	0.43

### PCA of stomatal and photosynthetic features from two sites

**Figure 5.**
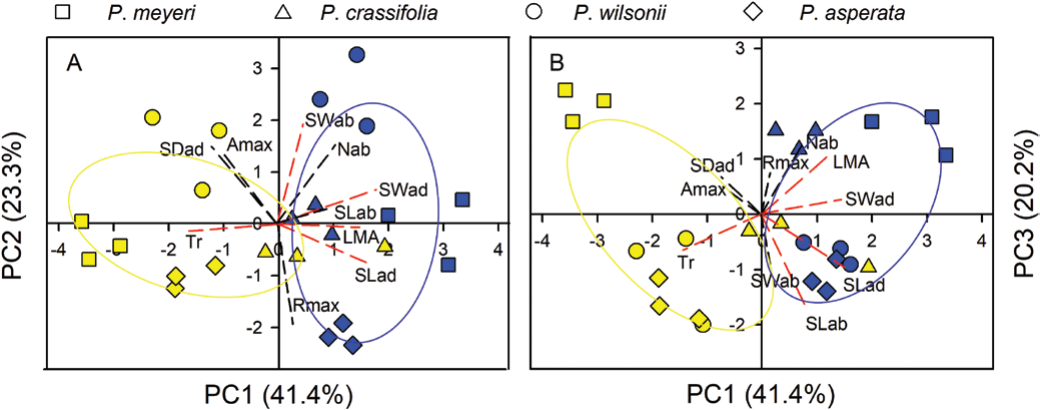
Results of PCA of stomatal density (SD), number of stomatal rows (N), stomatal length (SL) and stomatal width (SW), measured on the adaxial (ad) and abaxial (ab) surface of the leaf. The analysis also includes leaf mass per area (LMA) and photosynthetic features: net photosynthetic rate (Pn); transpiration rate (Tr); respiration rate during daytime darkness (Rday). Individuals of each species are shown with different symbols: *P*. *meyeri* (square), *P*. *asperata* (diamond), *P*. *crassifolia* (triangle) and *P*. *wilsonii* (circle). Species grown in WCG and DCG are shown in blue and yellow, respectively. Red intermittent lines are for the major loadings to the axis. The first component explains 41.4 % of the variation in the data set, the second component explains 23.3 % (A) and the third component explains 20.2 % (B).

A PCA was performed to reveal the relationships between traits in each species when grown at the arid and humid sites. Three axes were identified (each with an eigenvalue > 1) that together explained 84.9 % of the total variation at the two sites (Fig. 5). Due to similarity between the proportion of variation explained by axis 2 (23.3 %) and axis 3 (20.2 %), two graphs were produced to show the relationships (Fig. 5A and B). Apparently, species in the arid and humid gardens were separated along principle axis 1 (PC1) (Fig. 5). For axis 1, SL and SW on the adaxial surface, Tr and LMA contributed greatly to the distribution, while SL and SW on the abaxial surface accounted more for the distributions on axis 2 and axis 3, respectively **[see****Supporting Information—Table S2****]**.

## Discussion

Transplanting saplings into humid and arid common gardens can simulate a climate change scenario (Gavazov *et al.* 2013; Cárcer *et al.* 2017). A previous study suggested that patterns of stomatal distribution on the two leaf surfaces generally change with the environment (Fraser *et al.* 2009), and stomata can operate independently across and between surfaces of dorsiventral leaves (Fanourakis *et al.* 2015; Richardson *et al.* 2017). We found that there were significant differences on both the adaxial and abaxial surface between every species in the two common gardens (Figs 2 and 3). Research examining the adaxial and abaxial surface of conifers has shown that a reduction in stomatal density on both surfaces and reduced stomatal conductance may have important consequences for the response of pine trees to water stress during long-term growth at elevated CO_2_ concentrations (Pearson *et al.* 1995; Lin *et al.* 2001). Our result, however, was different, suggesting that, during long-term growth under drought conditions, stomatal density remained constant while stomatal conductance decreased (Figs 2 and 3). This is consistent with data on *Pinus edulis* in a 7-year experiment (Guérin *et al.* 2018) and with previous reports suggesting that physiological responses tend to be stronger and faster than morphological ones (Valladares *et al.* 2000; Hodge 2004; Wyka *et al.* 2007, 2012; Grossiord *et al.* 2017a, b). Wyka *et al.* (2012) attributed this to the structural demands on conifers’ leaves resulting from their perennial life cycle, and also to the ghosts of this lineage’s evolutionary past. Thus, physiological processes such as the rate of photosynthesis and stomatal conductance were generally highly plastic (Fig. 3A and B). This plasticity may function as an adaptive mechanism: unlike morphological traits, photosynthetic features can be quickly restored to their original state when extreme conditions no longer prevail, without requiring tissue reconstruction or substantial maintenance costs (Metlen *et al.* 2009).

Leaf gas exchange capacity depends on both stomatal traits (notably the number and/or size of the stomata) and their behaviour (Hetherington and Woodward 2003; Zwieniecki *et al.* 2016). Although there was no further analysis of non-independent traits, their physiological significance could not be ignored. In our research, we noticed that iWUE did not differ significantly between WCG and DCG, whilst the P/R for all species except *P*. *crassifolia* was different between the common gardens (Fig. 3E and F). For *P*. *crassifolia*, the reason may be the greater decline of Tr at the arid site to prevent water loss, representing a positive response to drought stress. For the other three species, P/R decreased no matter whether Pn, Gs and Tr increased or not from WCG to DCG (Fig. 3). Similar examples can be found in *P. edulis* and *Juniperus monosperma* which, when grown with reduced levels of precipitation, exhibit lower rates of photosynthesis and stomatal conductance together with increased water use efficiency (Grossiord *et al.* 2017b). Stomatal pore index (SI) and its relationship with total leaf area is also important with respect to gas exchange (Zwieniecki *et al.* 2016). Our results revealed that changes in SL, SW and S were not associated with much change in SI, which could help us to understand why photosynthetic traits changed in the ways described above.

Most previous research about the plasticity in traits related to species’ original habitats has been based on experiments comparing plants grown under controlled conditions, such as paired shaded and full-sunlight plots (Moreira *et al.* 2013). In the wild, we expect plasticity to be influenced by both precipitation (the main factor in this study) and other environmental factors. Valladares *et al.* (2007), indeed, argued that ecological limits resulting from abiotic and biotic factors would influence phenotypic plasticity in unpredictable ways. However, in our study, species that originated from arid habitats exhibited non-significant plasticity compared to those from humid habitats (Table 3), although species grown in the two common gardens did differ (Fig. 5). The main trait variations in each species are revealed by the direction of change in the graph. It appears that each species exhibits a different strategy when modifying traits to respond to growing in a dry environment. This result suggests that the phenotypic plasticity of these four species was site-specific rather than, as we first expected, associated with their original habitats. In fact, *P*. *crassifolia* (arid habitat) and *P*. *asperata* (humid habitat) did respond in the way we expected: *P*. *crassifolia* reduced water loss and enhanced iWUE while *P*. *asperata* maintained high photosynthetic capacity but with high respiration (Fig. 3). Thus, our results indicate that *P*. *crassifolia* may survive well when suffering long-term water limitation by enhancing iWUE without reducing P/R (Fig. 3E and F). It was, however, also limited by traits other than these, which may be why Valladares *et al.* (2007) proposed ecological limits to plant phenotypic plasticity and Nicotra *et al.* (2010) aimed to synthesize multiple factors to examine the potential roles of plasticity in determining plant response to and effects of climate change. Therefore, long-term observation and molecular evidence (e.g. gene differential expression) of phenotypes could provide more substantive evidence explaining this hypothesis (Matesanz *et al.* 2010).

## Conclusions

High resolution sampling of adaxial and abaxial leaf surfaces combined with direct measurement of carbon acquisition, water use and gas exchange in four *Picea* species provided a new insight into the plasticity of stomatal density, size and photosynthetic performance in the leaves of individuals growing in two regions with different levels of precipitation. The four species in the same genus analysed in this study exhibited comparatively high photosynthetic plasticity and low stomatal plasticity in the two common gardens. The differences in the plasticity of the independent traits resulted in divergence of the iWUE and P/R between species when comparing the results obtained in the arid and humid gardens. This could be expected, in turn, to affect the species’ relative capacities for rates of water loss and carbon accumulation. Our results also indicated that site-specific conditions can mask habitat variations, and thus should always be considered in studies of habitat trends in the future. Finally, we can make a preliminary suggestion that *P*. *crassifolia* should survive best because of its ability to enhance iWUE and keep P/R unchanged during long-term drought stress, while the other species slow down physiological activities to acclimate to drought stress. This result may be useful when planning plant reforestation efforts. In the future, synthesizing multiple factors to examine how climate change affects the potential roles of plasticity in determining plant responses would be valuable.

## Sources of Funding

This research was supported by the National Natural Science Foundation of China (grant nos. 31522013 and 31370603) and Major Special Science and Technology Project of Gansu Province (18ZD2FA009).

## Contributions by the Authors

C.M.Z., M.H.W. and J.R.W. conceived and designed the experiment. M.H.W. and A.P.Z. performed the experiment and analysed data with the help of X.W.Z. M.H.W. wrote the manuscript. S.S. and C.M.Z. discussed and revised the manuscript.

## Conflict of Interest

None declared.

## Supporting Information

The following additional information is available in the online version of this article—

Table S1. Analysis of Pearson’s correlation between stomatal and photosynthetic traits.

Table S2. Values of loadings and their cumulative proportions for the four species in the principal component analysis.

## Supplementary Material

plz034_suppl_Supplementary_Table_1

plz034_suppl_Supplementary_Table_2
