# Supplementary material for: Phenotypic plasticity of stomatal and photosynthetic features of four Picea species in two contrasting common gardens
Source: AoB Plants. 2019 Jun 8;11(4):plz034. doi: 10.1093/aobpla/plz034 (PMC6621916; doi:10.1093/aobpla/plz034)
Supplement: plz034_suppl_Supplementary_Table_1 [file plz034_suppl_supplementary_table_1.docx]

Table S1 Analysis of Pearson’s correlation between stomatal and photosynthetic features.

| Correlation | Nad | SLad | SWad | SDab | Nab | SLab | SWab | Pn | Gs | Tr | Rday | LMA |
| --- | --- | --- | --- | --- | --- | --- | --- | --- | --- | --- | --- | --- |
| SDad | 0.510* | -0.759** | -0.335 | 0.431* | 0.188 | -0.388 | 0.225 | 0.324 | 0.224 | 0.263 | -0.11 | -0.28 |
| Nad |  | -0.105 | 0.403 | 0.719** | **0.803**** | -0.001 | 0.478* | 0.169 | -0.018 | -0.470* | -0.166 | 0.358 |
| SLad |  |  | 0.626** | -0.228 | 0.136 | 0.658** | 0.129 | -0.472* | -0.419* | -0.516** | 0.078 | 0.494* |
| SWad |  |  |  | 0.212 | 0.603** | 0.349 | 0.249 | -0.373 | -0.392 | -0.723** | -0.181 | 0.712** |
| SDab |  |  |  |  | **0.811**** | -0.204 | 0.401 | 0.490* | 0.375 | -0.321 | -0.325 | 0.216 |
| Nab |  |  |  |  |  | -0.056 | 0.29 | 0.2 | 0.108 | -0.610** | -0.126 | 0.669** |
| SLab |  |  |  |  |  |  | 0.567** | -0.279 | -0.366 | -0.265 | -0.155 | 0.014 |
| SWab |  |  |  |  |  |  |  | 0.169 | 0.048 | -0.064 | -0.549** | -0.159 |
| Pn |  |  |  |  |  |  |  |  | **0.829**** | 0.35 | -0.225 | -0.18 |
| Gs |  |  |  |  |  |  |  |  |  | 0.545** | -0.254 | -0.22 |
| Tr |  |  |  |  |  |  |  |  |  |  | -0.189 | -0.681** |
| Rday |  |  |  |  |  |  |  |  |  |  |  | 0.224 |

ad indicates adaxial and ab indicates abaxial. Correlation coefficients > 0.8 are highlighted in bold. When we conducted the principal component analysis, Nad and SDab were replaced by Nab and Gs was replaced by Pn due to their high correlation.

* Correlation is significant at the 0.05 level (2-tailed).

** Correlation is significant at the 0.01 level (2-tailed).
