# Supplementary material for: Phenotypic plasticity of stomatal and photosynthetic features of four Picea species in two contrasting common gardens
Source: AoB Plants. 2019 Jun 8;11(4):plz034. doi: 10.1093/aobpla/plz034 (PMC6621916; doi:10.1093/aobpla/plz034)
Supplement: plz034_suppl_Supplementary_Table_2 [file plz034_suppl_supplementary_table_2.docx]

Table S2 Values of loadings and their cumulative proportions for the four species in the principal component analysis.

| Traits | PC 1 | PC 2 | PC 3 |
| --- | --- | --- | --- |
| SDad | -0.59 | 0.49 | 0.36 |
| SLad | **0.87** | -0.20 | -0.41 |
| SWad | **0.90** | 0.25 | 0.10 |
| Nab | 0.52 | 0.59 | 0.58 |
| SLab | 0.50 | 0.17 | **-0.80** |
| SWab | 0.13 | **0.81** | -0.48 |
| Pn | -0.48 | 0.59 | 0.18 |
| Tr | **-0.89** | -0.17 | -0.30 |
| Rday | 0.17 | -0.76 | 0.34 |
| LMA | **0.80** | 0.00 | 0.51 |
| Cumulative proportion (%) | 41.4 | 64.7 | 84.9 |

ad indicates adaxial and ab indicates abaxial. The values of loadings > 0.8 are highlighted in bold.
